# *Bacillus velezensis* CLA178-Induced Systemic Resistance of *Rosa multiflora* Against Crown Gall Disease

**DOI:** 10.3389/fmicb.2020.587667

**Published:** 2020-10-22

**Authors:** Lin Chen, Xinghong Wang, Qinghua Ma, Lusen Bian, Xue Liu, Yan Xu, Huihui Zhang, Jiahui Shao, Yunpeng Liu

**Affiliations:** ^1^Experimental Center of Forestry in North China, Chinese Academy of Forestry, Beijing, China; ^2^Key Laboratory of Agricultural Microbial Resources Collection and Preservation, Ministry of Agriculture and Rural Affairs, Institute of Agricultural Resources and Regional Planning, Chinese Academy of Agricultural Sciences, Beijing, China; ^3^Jiangsu Provincial Key Lab for Organic Solid Waste Utilization, National Engineering Research Center for Organic-based Fertilizers, Nanjing Agricultural University, Nanjing, China

**Keywords:** induced systemic resistance, plant growth-promoting rhizobacteria, rose, crown gall disease, hormone

## Abstract

Plant growth-promoting rhizobacteria (PGPRs) are able to activate induced systemic resistance (ISR) of the plants against phytopathogens. However, whether and how ISR can be activated by PGPRs in plants of the *Rosa* genus is unclear. The effects of PGPR *Bacillus velezensis* CLA178 and the pathogen *Agrobacterium tumefaciens* C58 on the growth, plant defense-related genes, hormones, and reactive oxygen species (ROS) in the rose plants were compared. Pretreatment with CLA178 significantly reduced crown gall tumor biomass and relieved the negative effects of the C58 pathogen on plant biomass, chlorophyll content, and photosynthesis of roses. Pretreatment of the roots with CLA178 activated ISR and significantly reduced disease severity. Pretreatment with CLA178 enhanced plant defense response to C58, including the accumulation of ROS, antioxidants, and plant hormones. Moreover, pretreatment with CLA178 enhanced C58-dependent induction of the expression of the genes related to the salicylic acid (SA) or ethylene (ET) signaling pathways. This result suggested that SA- and ET-signaling may participate in CLA178-mediated ISR in roses. Additional experiments in the Arabidopsis mutants showed that CLA178 triggered ISR against C58 in the *pad4* and *jar1* mutants and not in the *etr1* and *npr1* mutants. The ISR phenotypes of the Arabidopsis mutants indicated that CLA178-mediated ISR is dependent on the ET-signaling pathway in an NPR1-dependent manner. Overall, this study provides useful information to expand the application of PGPRs to protect the plants of the *Rosa* genus from phytopathogens.

## Introduction

The *Rosa* genus consists of woody plants that are grown worldwide due to their importance in horticulture, cosmetics, and medicine (Hassanein, 2010; Nadeem et al., 2015). This genus includes approximately 200 species and 20,000 cultivars. Roses are typical ornamental plants and have been developed as garden plants or for the cut rose market. Rose hips are used in food and medical applications; rose flowers are also cultivated for use in food and rose oil production (Byrne, 2009). However, most rose species are susceptible to crown gall disease caused by pathogenic *Agrobacterium* strains, such as *Agrobacterium tumefaciens* (other names: *Agrobacterium fabrum* or *Rhizobium radiobacter*; Martí et al., 1999; Gan and Savka, 2018; Diel et al., 2019). Rose plants infected by *A. tumefaciens* develop crown galls on the basal portions of their stems and roots leading to reduced plant growth. Crown gall disease impairs nutrient uptake, growth, and production. Severe disease can cause death of the plants and serious economic losses (López-López et al., 1999).

Plant diseases can be reduced by several methods, such as application of chemical agents, transgenic approaches, and biological control by the beneficial bacteria (Waard et al., 1993; Dong et al., 2007; Liu et al., 2017). The biological control method involving beneficial rhizobacteria is advantageous for protection of the plants from pathogen attack due to environmental safety. Plant growth-promoting rhizobacteria (PGPRs) benefit plants by improving nutrient uptake, promoting plant growth, antagonizing soilborne pathogens, and enhancing plant resistance (Durán et al., 2018; Stringlis et al., 2018b; Pascale et al., 2020). Biocontrol using PGPR strains has been studied in detail in agricultural crops, such as cucumber, maize, wheat, soybean, lettuce, and barley; however, the application of PGPRs in woody plants has not been well developed, and available information on the subject is considerably lacking (Pieterse et al., 2014; Berendsen et al., 2018).

PGPR can prime the plant immune system for rapid response to a broad range of pathogens without direct contact with the pathogens (Glazebrook, 2005; Yi et al., 2013; Stringlis et al., 2018a). This type of resistance is called induced systemic resistance (ISR). Induction of ISR is an efficient means of biocontrol by PGPRs. ISR is long-lasting and continuously protects the plants (Pieterse et al., 2014).

The mechanism of the onset of ISR triggered by PGPR is incompletely understood; however, several stimulators have been proposed, such as flagellin, lipopolysaccharides (LPS), volatile organic compounds (VOCs), and siderophores (Romera et al., 2019). In plants, jasmonic acid (JA)/ethylene (ET) signaling pathways are important for the activation of ISR by PGPRs (Glazebrook et al., 2003; Pieterse et al., 2014). However, in some cases, salicylic acid (SA) signaling pathway is also involved in ISR. For example, ISR in Arabidopsis triggered by *B. cereus* AR156 requires JA/ET and SA signaling pathways (Niu et al., 2011). Most of the studies on ISR were performed in Arabidopsis or crops. However, the signaling pathways involved in ISR may differ between various plant species and microbes (Romera et al., 2019).

*Bacillus* species are the most widely used PGPR strains for promotion of plant growth and protection of the plants against biotic and abiotic stresses due to their stress tolerance (Nicholson, 2002; Borriss, 2011). *Bacillus velezensis* CLA178 is a beneficial bacterium isolated from the rhizosphere soil of *Rosa multiflora* that can negatively influence the C58 pathogen infection in plants. In this study, CLA178 was shown to activate ISR against crown gall disease in rose. Physiological analysis and evaluation of the expression of the genes related to plant defense in rose were performed in addition to comparison of ISR phenotypes of various Arabidopsis mutants. These results provided insight into the induction process.

## Materials and Methods

### Isolation and Identification of *B. velezensis* CLA178

*Bacillus velezensis* CLA178 was isolated from the rhizosphere soil of *Rosa multiflora*. Its morphological characteristics were observed on Luria-Bertani (LB) medium (5 g l^–1^ yeast extract, 10 g l^–1^ tryptone, 10 g l^–1^ NaCl; pH 7.0–7.2) agar plates. The 16S rRNA gene of CLA178 was amplified from the CLA178 genome with the universal primers 27F and 1492R. The 16S rRNA gene sequence and genome sequence of the CLA178 strain were submitted to the NCBI GenBank.

The housekeeping gene *rpoB* of CLA178 was compared with the sequences available in the NCBI GenBank. Multiple alignments were performed by CLUSTAL_X software. The phylogenetic trees were constructed with the MEGA 7 software.

### Genome Sequencing and Genotypic Characterization

The complete genome sequencing of the CLA178 strain was performed by combining and Illumina MiSeq system and the PacBio RSII high-throughput sequencing technology at Shanghai Personal Biotechnology Co., Ltd., China. The raw data were filtered and trimmed by AdapterRemoval (ver. 2.1.7) and SOAPec (ver. 2.0) (Luo et al., 2012; Schubert et al., 2016). The reads of Illumina MiSeq system were assembled using A5-miseq (ver. 20160825) and SPAdes genome assembler (ver. 3.11.1) with default parameters (Bankevich et al., 2012; Tritt et al., 2012). The reads of PacBio RSII were assembled into contigs using HGAP4 and CANU (Chin et al., 2016; Koren et al., 2017). The contigs obtained by Illumina MiSeq system and PacBio RSII were analyzed collinearly using MUMmer (Delcher et al., 1999). The quality of genome assembly was improved by the Pilon software (Walker et al., 2014).

The relatedness of the genome sequence of CLA178 to the complete genome sequences of related strains was determined based on the average nucleotide identities (ANI). Genome sequences in a pairwise comparison were split into 1,000 bp windows and aligned with nucmer in MUMmer v3.23 (ANIm) (Kurtz et al., 2004). ANI were calculated using JSpecies v1.2.1 (Meier-Kolthoff et al., 2014).

### Plant and Growth Conditions

*Rosa multiflora* ‘Innermis’ stems were surface-sterilized with 75% (vol:vol) ethanol and then with 2% (vol:vol) NaClO. The surface-sterilized stems were cut into segments and grown in sterile vermiculite with rooting powder or in 1/4 MS media containing 3% sucrose, 0.6% agar, 0.5 mg l^–1^ 6-benzylaminopurine (6-BA), and 0.2 mg l^–1^ naphthaleneacetic acid. *Rosa multiflora* was cultivated at 25°C with a 14 h/10 h light/dark photoperiod.

Seeds of *Arabidopsis thaliana* ecotype Columbia (Col-0) and the *pad4* (Glazebrook et al., 1996), *jar1* (Staswick et al., 1992), *etr1* (Bleecker et al., 1988), and *npr1* (Cao et al., 1997) mutants were sown in sterile vermiculite. Thirty-day-old seedlings were used for the experiments. *Arabidopsis thaliana* (Arabidopsis) plants were cultivated in a growth chamber at 25°C with a photoperiod of 14 h of light and 10 h of darkness.

### Strain Cultivation and Inoculation

*Bacillus velezensis* CLA178 and *Agrobacterium tumefaciens* C58 (other names: *Agrobacterium fabrum*, *Rhizobium radiobacter*; ATCC 33970^*T*^ = ACCC 10055^*T*^; Martí et al., 1999) were cultured at 30°C with shaking at 0.65 *g* (170 rpm, radium = 2 cm) for 10–12 h in LB medium. The cultures were then centrifuged and resuspended in sterile phosphate buffer (PBS, pH 7.0).

To measure the crown gall tumor of the plants, the seedlings were inoculated with PBS or *B. velezensis* CLA178 at a final density of 5 × 10^6^ CFU ml^–1^ medium. On the second day, the stem was infected with the pathogen *A. tumefaciens* C58 at a density of 10^9^ CFU ml^–1^ using a sterile needle (Song et al., 2015). Sterile PBS was used as a negative control. The rose and Arabidopsis seedlings were cultivated at 25°C with a 14 h/10 h light/dark photoperiod for 20 days and 14 days, respectively. The ratio of gall diameter/stem diameter (GD/SD), disease incidence, and disease index were calculated based on the analysis of 30 roses per site (five cuttings times 6 replicates per treatment) or 36 Arabidopsis plants per site (6 seedlings times 6 replicates per treatment). The disease index of rose crown gall disease was determined based on the following revised classification of Krastanova et al. (2010): 0 no galls; 1: small galls, 0 < GD/SD < 0.25; 2: medium galls, 0.25 < GD/SD < 0.75; 3: large galls, 0.75 < GD/SD < 1.25; 4: very large galls, GD/SD > 1.25. The tumor size of Arabidopsis was determined based on the following disease index: 0: no galls; 1: small galls, 0 < GD/SD < 1; 2: medium galls, 1 < GD/SD < 1.5; 3: large galls, 1.5 < GD/SD < 2; 4: very large galls, GD/SD > 2.

### Measurement of the Photosynthetic Rate and Chlorophyll

The photosynthetic rate was determined with a portable photosynthesis measurement system (Li-Cor-6400; Li-Cor Inc.). The chlorophyll content of fully expanded leaves was calculated with a chlorophyll meter (SPAD-502 Minolta). These measurements were calculated based on the analysis of six biological replicates.

### Measurement of Phytohormones, Reactive Oxygen Species (ROS) and Antioxidants

Surface-sterilized rose seedlings were cultured in a sterile triangular flask containing 1/4 MS medium, and 11-week-old seedlings were treated with CLA178 at a final density of 5 × 10^6^ CFUs ml^–1^ for one day. After infection with C58 for 6, 24, and 48 h, fresh plant stems (0.1 g) were collected and homogenized with 1 ml of PBS (pH 7.0). The homogenate was shaken at 4°C for 1 h and centrifuged. The supernatant was used to measure the content of SA, JA, ET, or ROS with an ELISA kit (Meimian Biotechnology Co., Ltd., Wu et al., 2018; Lin et al., 2020). The catalase and peroxidase activity were determined by the method reported by Chen et al. (2016). These measurements were analyzed based on six independent experiments.

### Transcription Analysis

The plant samples were flash-frozen in liquid nitrogen, and the RNA was extracted with a Qiagen RNeasy plant mini kit. The concentration and quality of the RNA were measured with a NanoDrop ND-2000 spectrophotometer. The transcript levels were determined by reverse transcription-polymerase chain reaction using a PrimeScript RT reagent kit (Takara Biotechnology Co.). Quantitative real-time polymerase chain reaction (qRT-PCR) was performed with TB Green^TM^ Premix EX Taq^TM^ (Takara) using an ABI Quantstudio^TM^ 3D digital PCR system (Life Technologies).

The transcription levels were measured using *RmACT* (*ACTIN*) as an internal reference. The following genes were assayed: *RmERF1* (*ETHYLENE-RESPONSIVE TRANSCRIPTION FACTOR 1*), *RmNPR1* (*NON-EXPRESSER OF PATHOGENESIS-RELATED GENES 1*), *RmAOS* (*ALLENE OXIDE SYNTHASE*), *RmMYC2* (encoding the transcription factor MYC2), and *RmPR1-4* (*PATHOGENESIS-RELATED PROTEIN 1-4*) with *RmPR2* encoding β-1,3-glucanase, *RmPR3* encoding basic chitinase, and *RmPR4* encoding a hevein-like protein. The primers for qRT-PCR are listed in Supplementary Table S1. The amino acid sequences of the selected genes from *Rosa multiflora* were aligned with the homologous genes from *Arabidopsis thaliana* (Supplementary Figure S1). The similarity of amino acid sequences of the selected genes from *R. multiflora* with the homologous genes from *A. thaliana* was analyzed (Supplementary Table S2). The specificity of the amplification was verified by melting-curve analysis and agarose gel electrophoresis. Relative transcription levels were calculated using the 2^–Δ^ ^Δ^ ^*CT*^ method based on three biological replicates (Livak and Schmittgen, 2001).

### Statistical Analysis

Differences between the treatments were statistically analyzed using analysis of variance (ANOVA) and Duncan’s multiple range tests (*P* < 0.05). SPSS version 25.0 was used for statistical analysis (SPSS Inc.).

## Results

### Identification of *B. velezensis* CLA178

**FIGURE 1:**
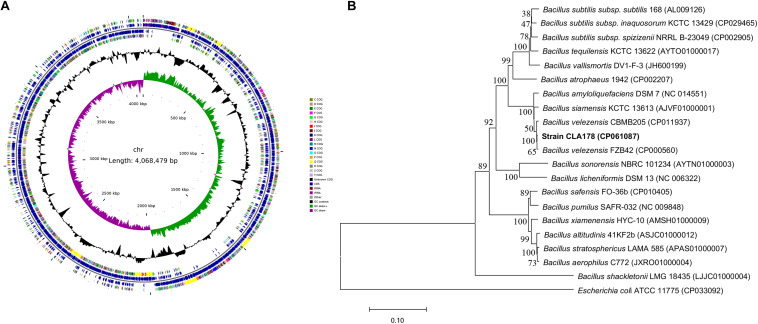
Genomic and phylogenetic structure characteristics of *Bacillus velezensis* CLA178. **(A)** Circular chromosome map of CLA178 was generated by CGView Server. **(B)** Neighbor-joining phylogenetic tree based on *rpoB* gene sequences showed the relationships between strain CLA178 and related taxa. Bootstrap values were determined based on 1000 replications. Bar, 0.1 substitutions per nucleotide position.

The strain CLA178 with antagonistic activities was isolated form the rhizosphere of healthy plants (*Rosa multiflora*) cultivated in the Fangshan (Beijing, China) nursery in soils that are known to be highly contaminated by pathogenic *A. tumefaciens*. The cells of the CLA178 strain were rod-shaped, motile, and Gram- positive and had the ability to form spores. The colonies on the LB agar were wrinkled. The CLA178 strain was able to grow in LB with 10% NaCl. The 16S rRNA and whole genome sequences of CLA178 were obtained and deposited in GenBank under the accession numbers MT071299 and CP061087, respectively. The circular chromosome map of CLA178 was presented in Figure 1A. Analyses of the GenBank and EzBioCloud databases revealed that the 16S rRNA gene sequence of CLA178 is closely related to *Bacillus* species. The phylogenetic analysis of the *rpoB* gene indicated that CLA178 belongs to *Bacillus velezensis* (Figure 1B). Additionally, the relatedness of the genome sequence of CLA178 to the genome sequence of related *Bacillus* species was determined based on ANI. The ANIm values of CLA178 to the type strain *B. velezensis* CBMB205 was 98.22%. Based on comparative analysis of the ANI values and phylogenetic analysis of *Bacillus* species, CLA178 was classified as *B. velezensis* (Kim et al., 2014; Miller et al., 2016; Fan et al., 2017; Rabbee et al., 2019).

### *B. velezensis* CLA178 Enhances Plant Biomass Under Crown Gall Disease Stress

**FIGURE 2:**
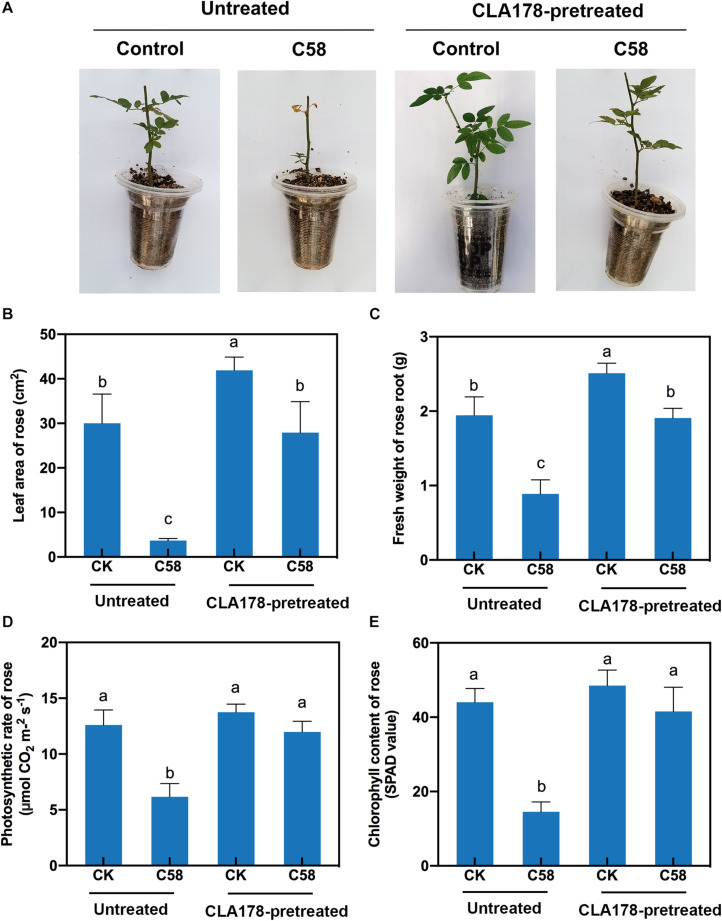
Effect of *Bacillus velezensis* CLA178 on biomass of rose under crown gall disease stress. **(A)** Representative image of rose inoculated with the strains for 30 days. The leaf area **(B)**, root fresh weight **(C)**, photosynthetic rate **(D)**, and chlorophyll content **(E)** of rose were determined. The rose plants were infected with C58 one day after inoculation with CLA178. The untreated and CLA178-pretreated rose plants treated with PBS (control) or infected with the C58 pathogen infection are shown. The values are the mean ± SD. Different letters above the bars indicate significant differences between the treatments (Duncan’s least significant difference, *P* < 0.05, *n* = 6).

To determine whether CLA178 is a PGPR, the impact of *B. velezensis* CLA178 on plant growth was measured. *B. velezensis* CLA178 enhanced leaf area and root biomass of rose plants indicating that CLA178 is the plant growth-promoting strain (Figures 2A–C). To assess the effect of *B. velezensis* CLA178 on growth of rose under crown gall disease stress, rose plants were preinoculated with CLA178 for one day in the rhizosphere before infection with *A. tumefaciens* C58. The indexes of plant growth and physiology were evaluated. The negative effect of infection of the stem by C58 on rose plants was evaluated at 30 days post inoculation (dpi). The results showed that the fresh root weight and leaf area of rose were significantly decreased after inoculation with *A. tumefaciens* C58 (Figures 2A–C). However, preinoculation with CLA178 before infection of the plant with C58 significantly reduced the negative effect of C58 on root biomass and leaf area (Figures 2A–C). Preinoculation with CLA178 also restored a reduction in chlorophyll and photosynthesis caused by C58 in rose (Figures 2D,E). These results indicate that CLA178 can suppress the negative effect of C58 on rose.

### *B. velezensis* CLA178 Induces Plant Resistance to *A. tumefaciens* C58 Infection

**FIGURE 3:**
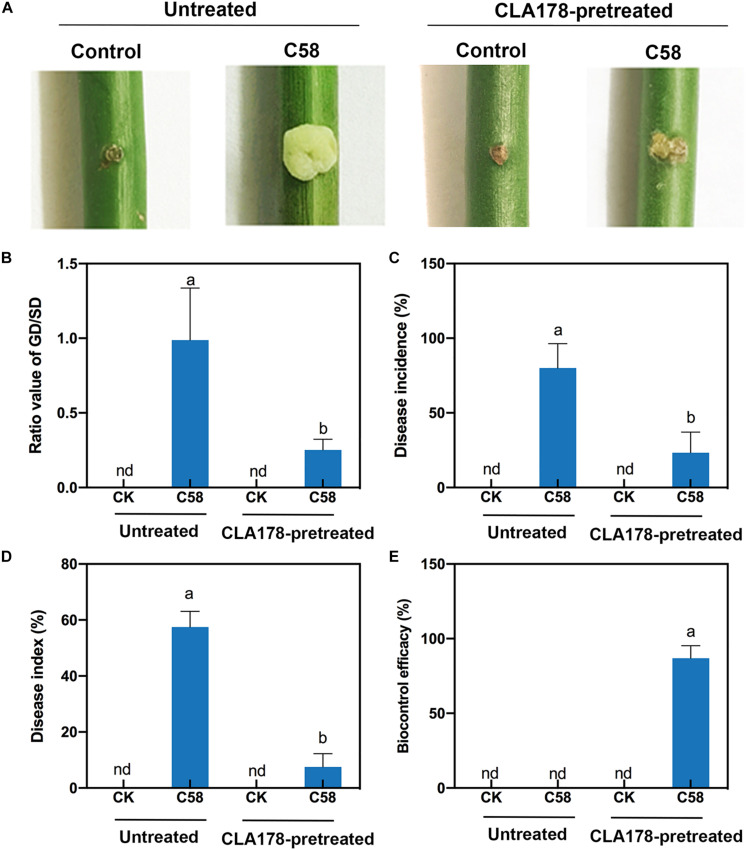
Effect of *Bacillus velezensis* CLA178 on crown gall tumor caused by *Agrobacterium tumefaciens* C58 in rose. **(A)** Representative image of rose inoculated with strains for 20 days. The GD/SD **(B)**, disease incidence **(C)**, disease index **(D)**, and biocontrol efficacy **(E)** in various treatments were measured in 30 plants. The rose plants were infected with C58 one day after inoculation with CLA178. The untreated and CLA178-pretreated rose plants with or without the C58 infection are shown. The values are the mean ± SD. nd, not detected. The same capital letter indicates the same index. Different lowercase letters of each index indicate statistically significant differences between the treatments (Duncan’s least significant difference, *P* < 0.05, *n* = 30).

To investigate whether *B. velezensis* CLA178 influences the interaction between rose and the pathogen to suppress the negative effect of C58 on the plants, the crown gall tumors of rose plants caused by C58 were evaluated. At 20 dpi with the *A. tumefaciens* C58 pathogen on the stem, rose plants pretreated with sterile PBS showed typical symptoms of crown gall tumors (Figure 3A). Preinoculation of CLA178 before the plant was infected with C58 resulted in a significant reduction in the GD/SD ratio relative to that in the C58 infection without preinoculation (0.25 vs. 0.99; Figure 3B). After plants were infected with C58, the disease incidence and disease index of the rose plants preinoculated with CLA178 were significantly lower than those in plants without preinoculation (disease incidence of 23.3 vs. 80% and disease index of 7.5 vs. 57.5%; Figures 3C,D). The biocontrol efficacy of CLA178 was 87% (Figure 3E). These results indicated that *B. velezensis* CLA178 can induce systemic resistance of rose against crown gall disease independently of direct contact with the pathogen.

### Reactive Oxygen Species Content and Antioxidant Activity Induced by the Strains

**FIGURE 4:**
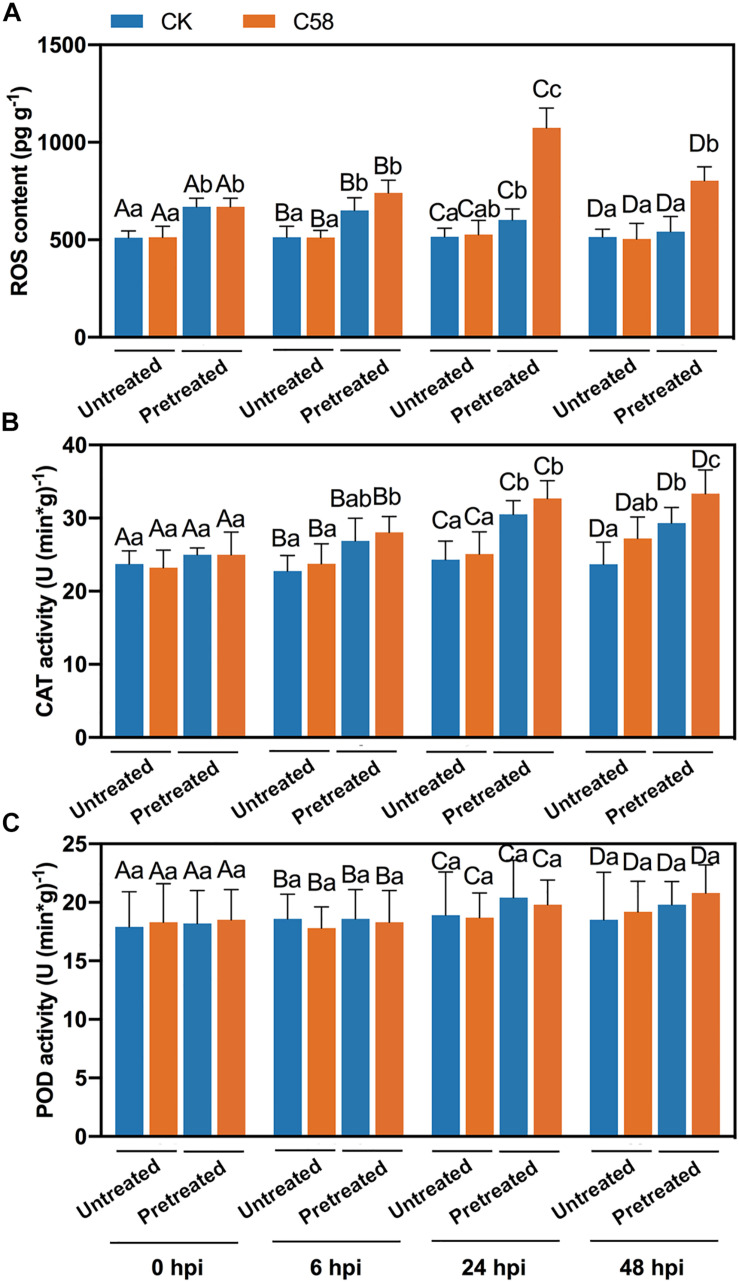
ROS content and CAT and POD activities in rose inoculated with CLA178, C58 or their combination. The plant tissues were harvested at 6, 24, and 48 hpi after *Agrobacterium* infection. ROS content **(A)** and CAT **(B)** and POD activities **(C)** in the plants were determined. “Untreated” and “Pretreated” indicate plants pretreated with PBS and CLA178, respectively. Sterile PBS was used as a control. The values are the mean ± SD. Capital letters indicate the grouping for statistical analysis. Different lowercase letters indicate statistically significant differences between the treatments (Duncan’s least significant difference, *P* < 0.05, *n* = 6).

To analyze the impact of preinoculation of CLA178 on rose resistance to the C58 pathogen, certain physiological indexes were determined. The accumulation of ROS is an important signal involved in the plant immune response (Rojas et al., 2014). Pretreatment with CLA178 enhanced the C58-induced ROS accumulation at 6, 24 and 48 h after C58 infection. Thus, pretreatment with CLA178 may enhance plant defense response when the plant was challenged with C58. Additionally, treatment with CLA178 without infection did not induce continuous ROS accumulation in the plant (Figure 4A).

Antioxidants are responsible for scavenging excessive ROS, and their activity always corresponds to the ROS content. The results of the assays of antioxidants were similar to the data obtained by the ROS accumulation assay. *Agrobacterium* infection alone induced only a slight increase in CAT activity at 48 h post infection. However, pretreatment with CLA178 significantly enhanced CAT activity at 6, 24 and 48 h after C58 infection (Figure 4B). Moreover, in plants pretreated with CLA178, the CAT activity was significantly increased upon C58 infection at 48 hpi (Figure 4B). The activity of POD was also determined, and no significant differences were observed between various treatments (Figure 4C). Overall, our results indicate that CLA178-primed rose plants have enhanced defense response to C58, including ROS accumulation and increased CAT activity.

### Phytohormones Induced by *B. velezensis* CLA178

**FIGURE 5:**
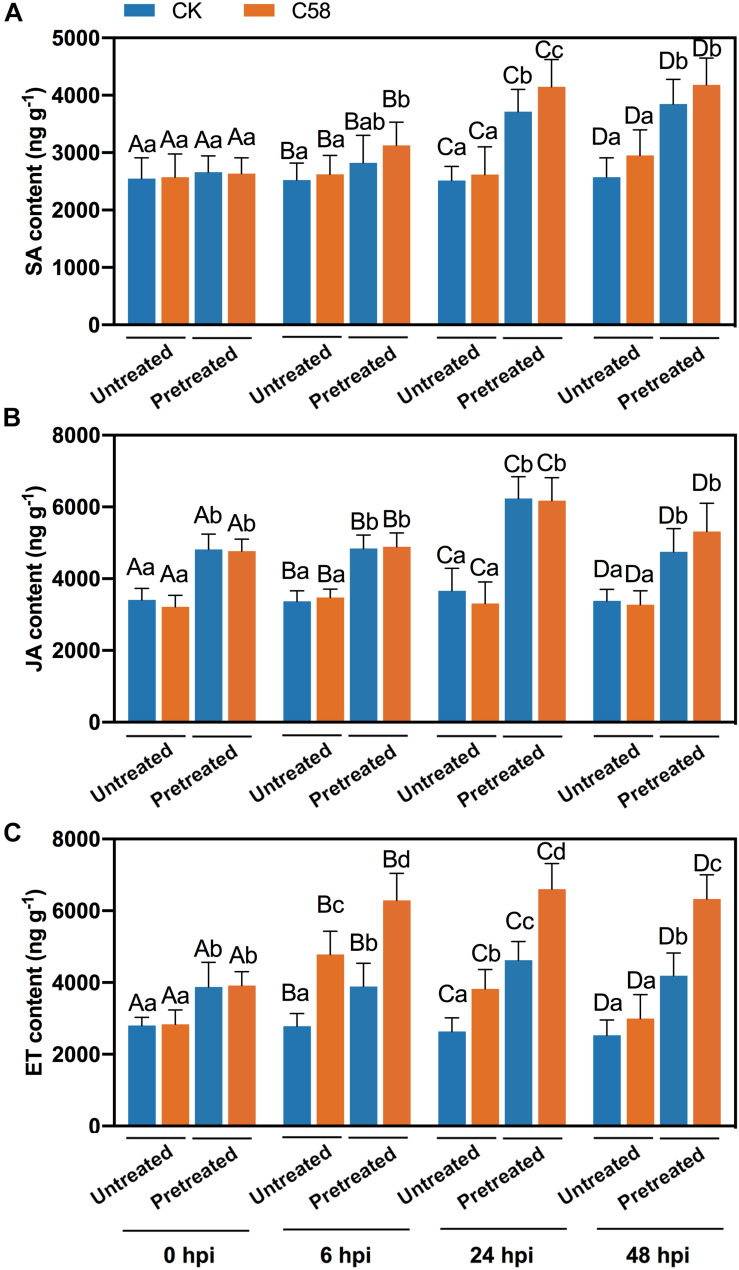
Phytohormone contents in rose. The plant tissues were harvested at 6, 24, and 48 hpi with C58. Salicylic acid **(A)**, jasmonic acid **(B)**, and ethylene contents **(C)** in rose were determined. Sterile PBS was used as a control. The values are the mean ± SD. Capital letters indicate the grouping for statistical analysis. Different lowercase letters indicate statistically significant differences between the plants subjected to various treatments (Duncan’s least significant difference, *P* < 0.05, *n* = 6).

The phytohormones SA, JA, and ET are involved in the defense responses and play important roles in the plant-microbe interactions. To investigate whether these phytohormones are involved in the defense response induced by CLA178, the levels of SA, JA and ET in the plants were measured. The contents of JA and SA in the CLA178-pretreatment group were significantly higher compared with those in the untreated group regardless of C58 infection; however, in CLA178-pretreated and untreated plants, C58 did not strongly influence the contents of JA and SA in the plants (Figures 5A,B). Infection with C58 increased the ET content at 6 h and 24 h after the infection in CLA178-pretreated and untreated plants. However, at 48 h after the infection, C58 induced ET accumulation only in CLA178-pretreated plants and not in the untreated plants (Figure 5C). This result indicates that ET may play an important role in CLA178-induced plant defense against pathogenic *Agrobacterium*.

### Defense-Related Genes Rby *B. velezensis* CLA178

**FIGURE 6:**
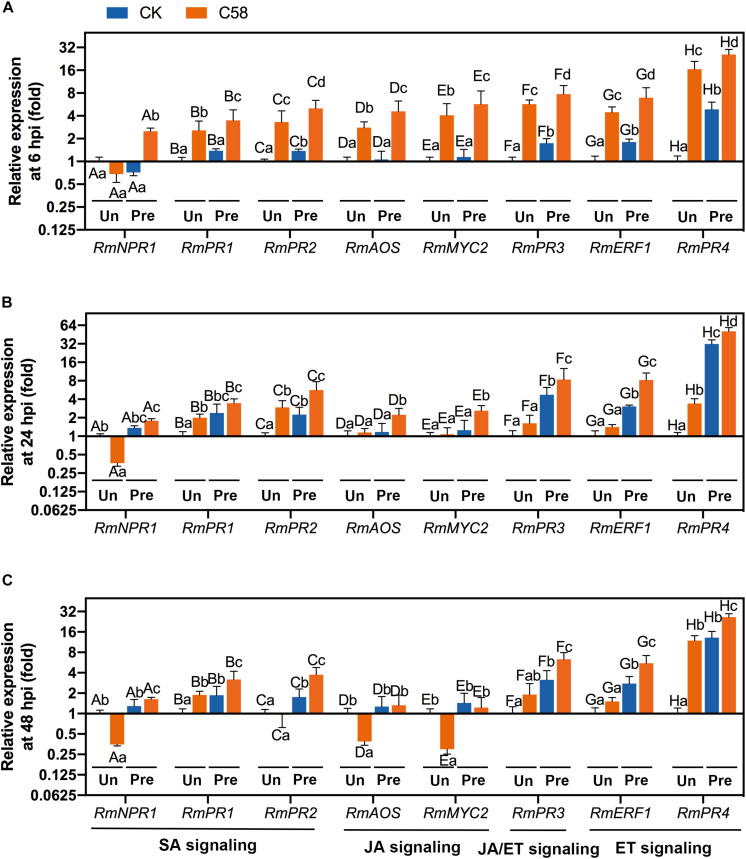
Expression of defense-related genes in rose. At 6 **(A)**, 24 **(B)**, and 48 hpi **(C)** with C58, plant samples were harvested for extraction of RNA. The values indicate the fold-change of the expression levels of each gene in the plants with inoculation relative to control detected by qRT-PCR. All genes were normalized using *ACTIN* as a reference. Expression levels of salicylic acid-related genes and ethylene- or jasmonic acid-related genes were determined in untreated and CLA178-pretreated rose plants treated with PBS or infected with the C58 pathogen. Control plants were treated with sterile PBS. “Un” and “Pre” indicate untreated and CLA178-pretreated plants, respectively. The data are shown as the mean ± SD (*n* = 3). Capital letters indicate the grouping for statistical analysis. Different lowercase letters indicate statistically significant differences between the plants subjected to various treatments (Duncan’s least significant difference, *P* < 0.05).

The SA, JA and ET-signaling pathways are important for ISR in the plants. To identify the pathway(s) regulated by CLA178, which may be responsible for CLA178-induced ISR, the transcription of plant genes involved in the SA-, ET- and JA-signaling pathways was measured using qRT-PCR. The descriptions of these genes are provided in Supplementary Table S1. *RmPR1* and *RmPR2* are involved in the SA-related pathway. *RmAOS* and *RmMYC2* are involved in the JA-related pathway. *RmERF1* and *RmPR4* are involved in the ET-related pathway. *RmPR3* is involved in the ET- and JA-related pathways. In plants infected with C58, the transcription of these genes was significantly upregulated by CLA178 pretreatment at 6, 24 and 48 hpi (Figures 6A–C). In untreated plants, the levels of upregulated genes induced by *Agrobacterium* infection at 6 hpi were higher than those at 24 and 48 hpi. In CLA178-pretreated plants, the transcription of the genes involved in the SA- and ET-related pathways was continuously upregulated at 6, 24, and 48 h post C58 infection; however, the transcription of *RmAOS* and *RmMYC2* involved in the JA-signaling pathway was not induced in plants infected with C58 at 48 hpi (Figures 6A–C). These data suggest that the induction of the genes of the SA- and ET-signaling pathways is involved in the CLA178-induced systemic resistance against C58.

### Signaling Pathway Dependence of *B. velezensis* CLA178-Mediated ISR

**FIGURE 7:**
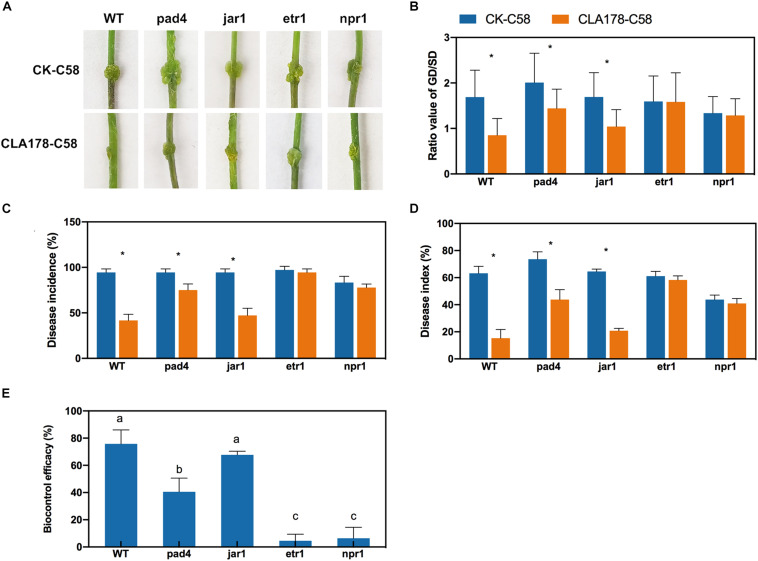
Protection induced by CLA178 against crown gall disease in wide-type and defense-related mutants (*pad4*, *jar1*, *etr1*, and *npr1*) in Arabidopsis. The 30-day-old seedlings were infected with C58 one day after inoculation with CLA178. **(A)** Symptom were observed 14 days after the C58 infection. **(B)** The GD/SD was measured. Disease incidence **(C)**, disease index **(D)**, and biocontrol efficacy **(E)** were calculated. Sterile PBS was used as a control. Asterisks indicate significant differences between the CLA178-treated samples and control according to Student’s *t*-test (*P* < 0.05, *n* = 36).

Then, we assessed whether blocking the signaling pathways disrupts the induction of ISR by CLA178. The resistance of wide-type Arabidopsis Col-0 (WT) and the defense-signaling mutants *pad4* (SA biosynthesis defective mutant phytoalexin deficient 4), *jar1* (JA response mutant), *etr1* (ET response mutant), and *npr1* (non-expresser of PR genes mutant) against C58 infection after induction by CLA178 was compared to confirm our findings. Preinoculation with CLA178 led to a significant reduction in the ratio of GD/SD, disease incidence, and disease index in WT, *pad4*, and *jar1*, but caused no significant reduction in these parameters in the *etr1* and *npr1* mutants at 14 dpi (Figure 7). The biocontrol efficacy of CLA178 against crown gall disease in WT and the *jar1* mutant was higher than that in *pad4* (Figure 7E). These results indicate that the ET-signaling pathway and NPR1 are necessary for the CLA178-induced ISR in the plants.

## Discussion

Crown gall disease is destructive to the production of many plant species of the *Rosaceae* family, such as cherry, peach, and pear trees (Gupta et al., 2010). PGPRs have been reported to protect woody plants from pathogen infection by direct antagonism; however, indirect protection of woody plant based on the induction of systemic resistance is poorly understood (Baltruschat et al., 2008; Compant et al., 2013). In this study, preinoculation with *B. velezensis* CLA178 induced rose resistance against the crown gall disease pathogen C58. Pretreatment with CLA178 enhanced an increase in ROS, SA, and ET contents upon C58 infection. The genes of the SA- or ET-signaling pathways were continuously induced by CLA178 pretreatment in rose plants after *Agrobacterium* infection. We hypothesized that CLA178 primes the rose plants for enhanced defense response to pathogenic *A. tumefaciens C58*, and the SA- and ET-signaling pathways may be involved in CLA178-induced ISR in rose. Subsequent experiments showed that CLA178 failed to induce the resistance against C58 in the *npr1* and *etr1* Arabidopsis mutants indicating that the ET-signaling pathway and NPR1 are necessary for CLA178-induced ISR against crown gall disease in Arabidopsis. This study may contribute to the biocontrol of crown gall disease in these plants.

PGPRs can promote rose growth (El-Deeb et al., 2012; Tariq et al., 2016); however, their biocontrol effect has not been evaluated in detail. *Rosa multiflora*, a typical species of the *Rosa* genus with high ornamental and economic value, often suffers from crown gall disease. Crown gall disease in other plants can be suppressed by *Agrobacterium rhizogenes* K84, *Agrobacterium vitis* VAR03-1, *Agrobacterium vitis* E26, *Rahnella aquatilis* HX2, etc., (Wang et al., 2003; Kawaguchi et al., 2008; Guo et al., 2009; Compant et al., 2013). However, most known biocontrol strains used to suppress crown galls are close relatives of the pathogenic “*Agrobacterium*” strains; thus, it is possible that non-pathogenic *Agrobacterium* biocontrol strains acquire virulence plasmids or produce them via a mutation (Mauck et al., 2010). This study is the first to demonstrate that *B. velezensis* CLA178 significantly reduces incidence of crown gall disease in rose by inducing ISR. Moreover, *B. velezensis* is non-pathogenic and environmentally safe to use than other closely related *Agrobacterium* species.

Plant immunity can be triggered by certain beneficial or pathogenic microbes. Oxidative burst is an early event that is always accompanied by MAMP-triggered immunity (MTI) or PAMP-triggered immunity (PTI) (Zamioudis and Pieterse, 2012). However, ROS accumulation in the plants was not increased by C58 in agreement with the data of some previous studies (Lee et al., 2009). In addition to a slight increase in CAT activity observed in plants at 48 hpi, CAT produced by C58 plays an important role in scavenging ROS produced by the plants in the early stage (Xu and Pan, 2000). The transcription of certain defense-related genes in the plants treated with C58 was minimized 24 hpi. The expression of these genes was suggested to be inhibited by T-DNA or vir proteins (Veena et al., 2003).

To investigate the molecular mechanisms of CLA178 induction of plant resistance to *A. tumefaciens* C58, the transcription of the genes involved in the SA-, JA-, and ET-signaling pathways was determined in rose, and the infection was assayed using related Arabidopsis mutants. The results indicate that the genes involved in the SA- or ET-signaling pathway were continuously induced by *Agrobacterium* in rose plants pretreated with CLA178; however, genes involved in the JA-signaling pathway were not induced. The investigation of gene transcription suggested that the SA- and ET- signaling pathways may be involved in ISR activated by *B. velezensis* CLA178 against crown gall disease in rose. The results obtained using various ISR phenotypes of the defense signaling mutants of Arabidopsis suggest that CLA178-induced ISR against crown gall disease in Arabidopsis is dependent on the ET-signaling pathway in an NPR1-dependent manner. PAD4 plays an important role in the SA-signaling pathway (Tsuda et al., 2008; Dempsey et al., 2011). CLA178 induces weaker ISR in the *pad4* mutant (Figure 7E). This result suggests that the SA-signaling pathway may be involved in CLA178-induced ISR.

Phytohormones can influence crown gall disease (Gohlke and Deeken, 2014). The SA content in rose was enhanced by PGPR CLA178 regardless of C58 infection; an increase in SA can repress the conjugal transfer of the Ti plasmid to reduce the virulence of C58 and modulate rhizosphere colonization by specific bacterial families to strengthen the plant immune system (Yuan et al., 2007; Lebeis et al., 2015). The accumulation of ET was observed in rose after CLA178 pretreatment or C58 infection, and ET accumulation was more intense in plants inoculated with a combination of CLA178 and C58. Upon the initiation of infection, ET in combination with indole acetic acid (IAA) is essential for growth of the tumors; however, ET suppresses the *vir* gene expression during the transformation (Lee et al., 2009; Gohlke and Deeken, 2014). The defense-related genes involved in the JA-signaling pathway were significantly influenced by infection with C58; however, the JA content of the rose plants was not significantly increased by C58 (Lee et al., 2009; Gohlke and Deeken, 2014; Song et al., 2015). Some studies demonstrated that the expression of the genes related to the hormone signaling and biosynthesis can be different, and the hormone signaling pathways can be activated by low levels of the hormones (Lee et al., 2009; Pieterse et al., 2014; Song et al., 2015; Wu et al., 2018). The JA content in rose was increased by CLA178 pretreatment; however, the expression of the genes involved in the JA-signaling pathway was not continuously induced by CLA178 in rose plants. Moreover, the *jar1* Arabidopsis mutant was still able to acquire CLA178-induced resistance. These results indicate that JA is not essential for ISR activated by CLA178. Moreover, the JA and ET contents in Arabidopsis leaves were not altered by PGPR *Pseudomonas fluorescens* WCS417r. WCS417r-mediated ISR in Arabidopsis depends on sensitivity to JA and ET (Pieterse et al., 2000). However, in this study, PGPR CLA178 enhanced the levels of these phytohormones in rose. Phytohormone accumulation was also observed in Arabidopsis treated with PGPR *B. amyloliquefaciens* SQR9 (Wu et al., 2018). The difference in the results may be due to different microbial and plant species.

In conclusion, *B. velezensis* CLA178 can suppress the negative effect of C58 on rose and induce systemic resistance against crown gall disease in Arabidopsis via the ET-signaling pathway in an NPR1-dependent manner. This study suggests that application of *B. velezensis* PGPR strains can be used to induce resistance against crown gall disease in woody plants in agroforestry production.

## Data Availability Statement

The datasets presented in this study can be found in online repositories. The names of the repository/repositories and accession number(s) can be found below: https://www.ncbi.nlm.nih.gov/, MT071299, https://www.ncbi.nlm.nih.gov/genbank/, CP061087.

## Author Contributions

LC, YL, and XW conceived and designed this research. LC, YX, LB, HZ, and XL conducted experiments. QM and JS analyzed data. LC and YL wrote the manuscript. All authors read and approved the manuscript.

## Conflict of Interest

The authors declare that the research was conducted in the absence of any commercial or financial relationships that could be construed as a potential conflict of interest.

## Supplementary Material

Supplementary Figure 1 Alignment of amino acid sequences of selected genes from *Rosa* multiflora with the homologous genes from *Arabidopsis thaliana*.

Supplementary Table 1 Dna primers used in this study.

Supplementary Table 2 The similarity of amino acid sequences of selected genes from *Rosa multiflora* with the homologous genes from *Arabidopsis thaliana*.
