# Supplementary material for: Bacillus velezensis CLA178-Induced Systemic Resistance of Rosa multiflora Against Crown Gall Disease
Source: Front Microbiol. 2020 Oct 22;11:587667. doi: 10.3389/fmicb.2020.587667 (PMC7642876; doi:10.3389/fmicb.2020.587667)
Supplement: Supplementary Figure 1 — Alignment of amino acid sequences of selected genes from Rosa multiflora with the homologous genes from Arabidopsis thaliana. [file Data_Sheet_1.docx]

**Supporting information**

**Fig. S1** Alignment of amino acid sequences of selected genes from *Rosa multiflora* with the homologous genes from *Arabidopsis thaliana.* (A) ERF1, (B) PR1, (C) PR2, (D) PR3, (E) PR4, (F) NPR1, (G) AOS, (H) MYC2


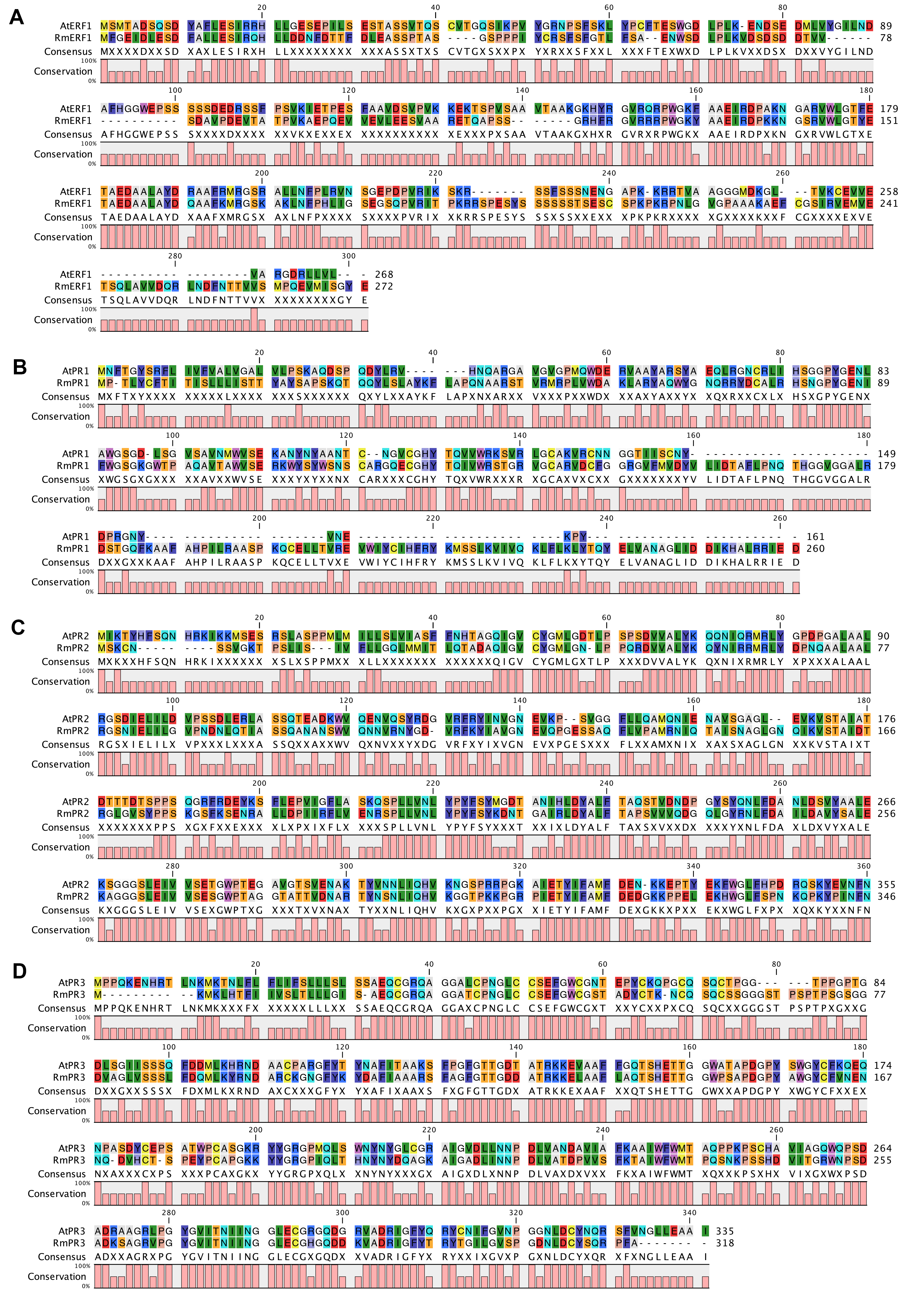


**Fig. S1** (continued from preceding page)


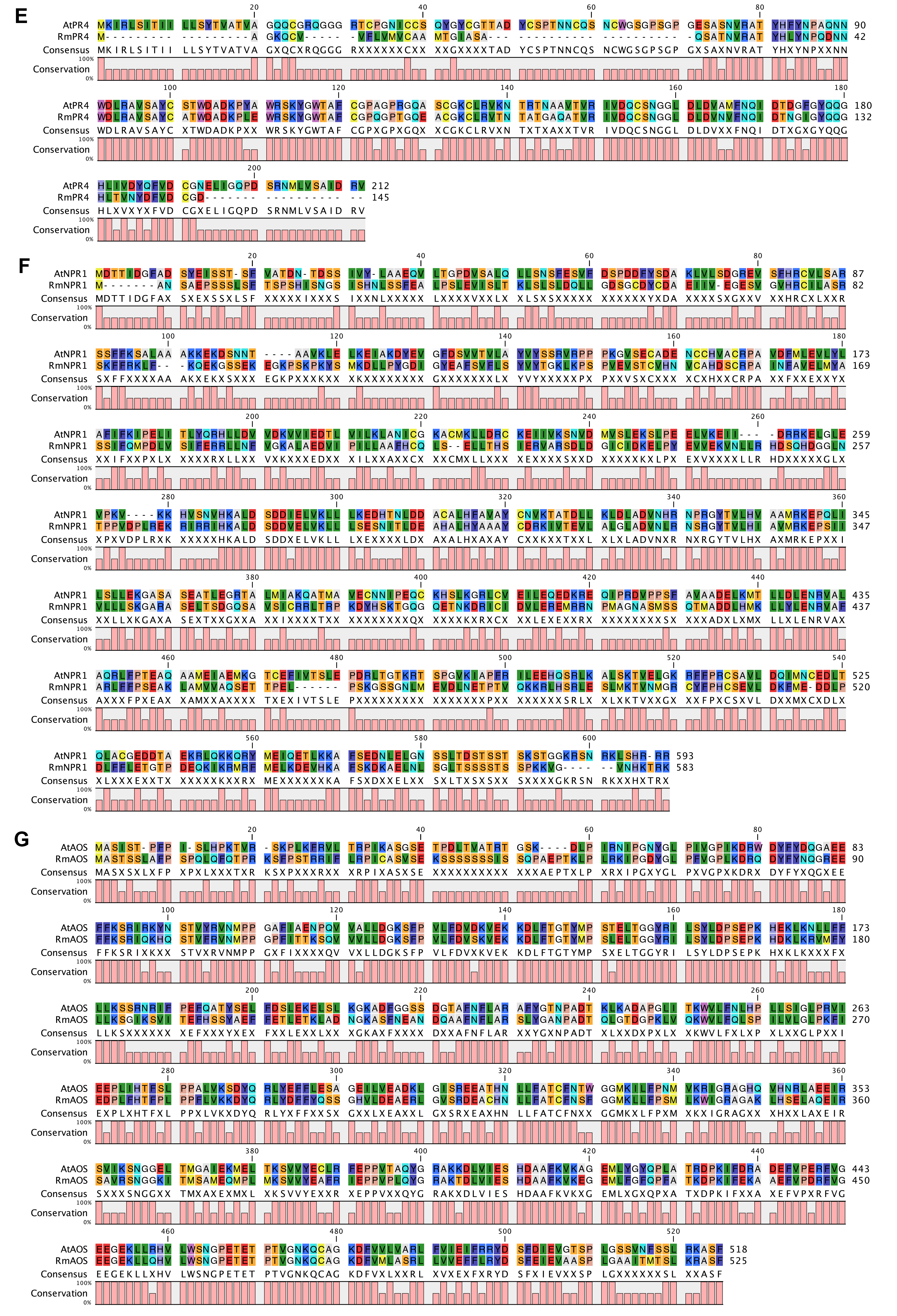


**Fig. S1** (continued from preceding page)


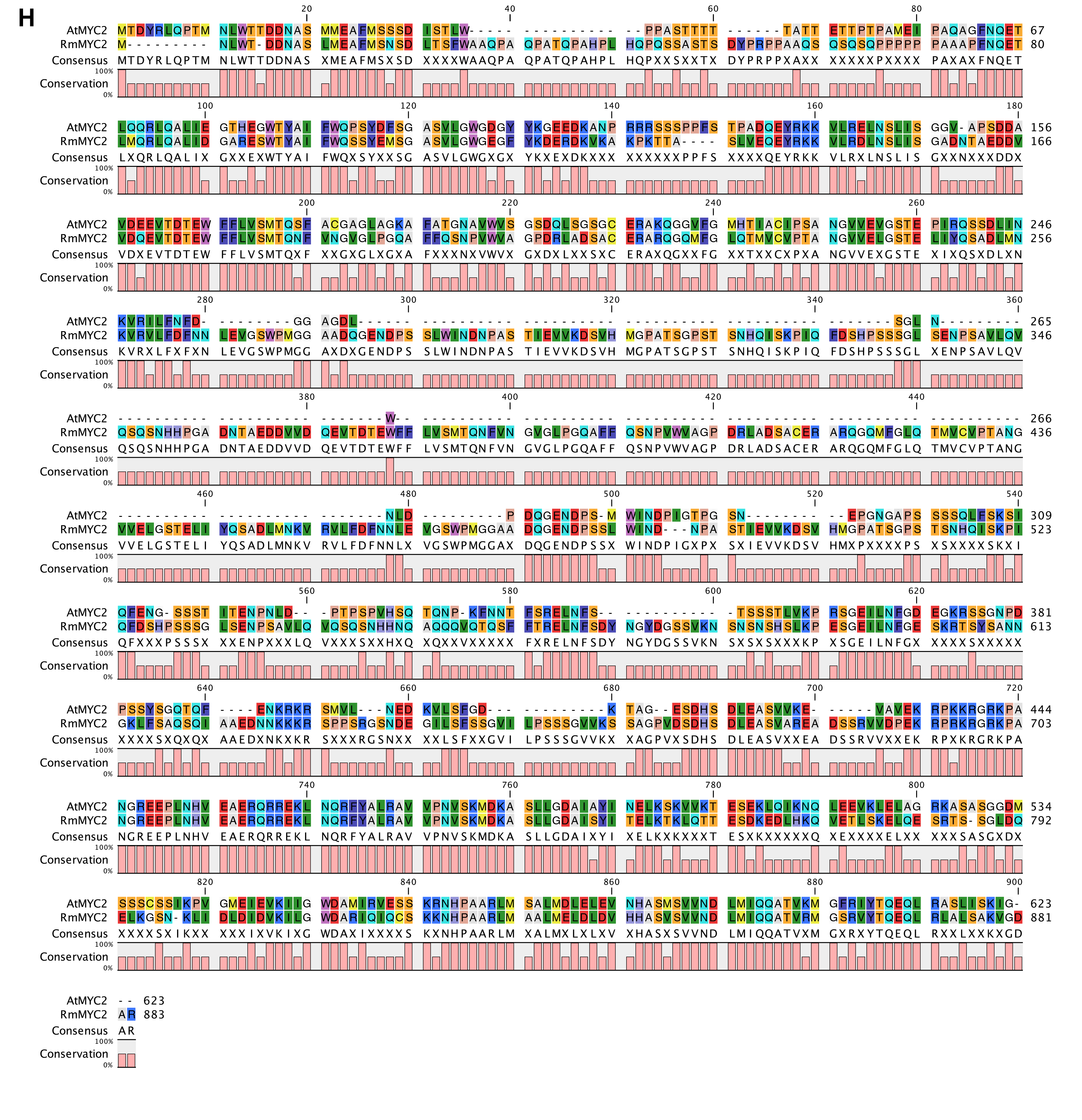


**Table S1.** DNA primers used in this study

| Gene name | Sequence (5'-3') | Gene location | Gene product |
| --- | --- | --- | --- |
| RmACT-F | TACAACTGGTATTGTCCTGGAC | Rmu_co8518979.1 | actin |
| RmACT-R | TCATGTCCCTGACTATTTCTCG |  |  |
| RmERF1-F | GATCGCAACCGGTTAGAATAAC | Rmu_sc0006452.1 | ethylene-responsive transcription factor 1 |
| RmERF1-R | CTCGTCTCAACCATCTCTACTC |  |  |
| RmPR1-F | CAGTGTGAACTTCTTACGGTTC | Rmu_sc0007727.1 | pathogenesis-related protein 1 |
| RmPR1-R | TTGCTACGAGTTCATACTGTGT |  |  |
| RmPR2-F | AATCAAAGTTTCCACAGCCATC | Rmu_sc0007855.1 | b-1,3-glucanase |
| RmPR2-R | GAAGAGAGCATAATCGAGACGA | |  |
| RmPR3-F | TGGGGTTATTGCTTTGTCAATG | Rmu_sc0008442.1 | basic chitinase |
| RmPR3-R | GGTTTGTTTGATTGTGGAGTCA |  |  |
| RmPR4-F | GGAAAACAATGCGTCGTGTTTT | Rmu_sc0000078.1 | hevein-like protein |
| RmPR4-R | TCTCACGTTAGTAGCACTTTGT |  |  |
| RmNPR1-F | CTTCGGAATTCTCGGGGTTATA | Rmu_sc0002759.1 | nonexpresser of PR genes 1 |
| RmNPR1-R | TCCGCATTTCTCTCTCTAGAAC |  |  |
| RmAOS-F | ACTACCAGAGACTCTACGACTT | Rmu_sc0001670.1 | allene oxide synthase |
| RmAOS-R | TGGGAAATAAAAGCTTCATGCC |  |  |
| RmMYC2-F | TTCAACCAGGAGACGCTTATG | Rmu_sc0002616.1 | transcription factor  MYC2 |
| RmMYC2-R | CGGAAATCAAAGAGTTGAGGTC | |  |
| 27F | AGAGTTTGATCCTGGCTCAG | | 16S rRNA |
| 1492R | GGTTACCTTGTTACGACTT | |  |

**Table S2.** The similarity of amino acid sequences of selected genes from *Rosa multiflora* with the homologous genes from *Arabidopsis thaliana*

| Gene name | Gene location | | Identify | Gene product | |
| --- | --- | --- | --- | --- | --- |
|  | *R. multiflora* | *A. thaliana* |  | |  |
| EFR1 | Rmu_sc0006452.1 | AT4G17500 | 42.16% | | ethylene-responsive transcription factor 1 |
| PR1 | Rmu_sc0007727.1 | AT2G14610 | 40.41% | | pathogenesis-related protein 1 |
| PR2 | [Rmu_sc0007855.1](https://www.rosaceae.org/jbrowse/index.html?data=data/rosa/rmultiflora_v1.0&loc=Rmu_sc0007855.1_g000007.1:-57..404&addFeatures=%5b%7b%22seq_id%22:%22Rmu_sc0007855.1_g000007.1%22,%22start%22:1,%22end%22:346,%22name%22:%22unnamed%20protein%20product%20Blast%20Hit%22,%22subfeatures%22:%5b%7b%22start%22:1,%22end%22:346,%22strand%22:%221%22,%22type%22:%22match_part%22%7d%5d%7d%5d&addTracks=%5b%7b%22label%22:%22blast%22,%22key%22:%22BLAST%20Result%22,%22type%22:%22JBrowse/View/Track/HTMLFeatures%22,%22store%22:%22url%22%7d%5d) | AT3G57260 | 59.94% | | b-1,3-glucanase |
| PR3 | [Rmu_sc0008442.1](https://www.rosaceae.org/jbrowse/index.html?data=data/rosa/rmultiflora_v1.0&loc=Rmu_sc0008442.1_g000009.1:-52..371&addFeatures=%5b%7b%22seq_id%22:%22Rmu_sc0008442.1_g000009.1%22,%22start%22:1,%22end%22:318,%22name%22:%22unnamed%20protein%20product%20Blast%20Hit%22,%22subfeatures%22:%5b%7b%22start%22:1,%22end%22:318,%22strand%22:%221%22,%22type%22:%22match_part%22%7d%5d%7d%5d&addTracks=%5b%7b%22label%22:%22blast%22,%22key%22:%22BLAST%20Result%22,%22type%22:%22JBrowse/View/Track/HTMLFeatures%22,%22store%22:%22url%22%7d%5d) | AT3G12500 | 68.11% | | basic chitinase |
| PR4 | [Rmu_sc0000078.1](https://www.rosaceae.org/jbrowse/index.html?data=data/rosa/rmultiflora_v1.0&loc=Rmu_sc0000078.1_g000011.1:0..166&addFeatures=%5b%7b%22seq_id%22:%22Rmu_sc0000078.1_g000011.1%22,%22start%22:21,%22end%22:145,%22name%22:%22unnamed%20protein%20product%20Blast%20Hit%22,%22subfeatures%22:%5b%7b%22start%22:21,%22end%22:145,%22strand%22:%221%22,%22type%22:%22match_part%22%7d%5d%7d%5d&addTracks=%5b%7b%22label%22:%22blast%22,%22key%22:%22BLAST%20Result%22,%22type%22:%22JBrowse/View/Track/HTMLFeatures%22,%22store%22:%22url%22%7d%5d) | AT3G04720 | 79.51% | | hevein-like protein |
| NPR1 | [Rmu_sc0002759.1](https://www.rosaceae.org/jbrowse/index.html?data=data/rosa/rmultiflora_v1.0&loc=Rmu_sc0002759.1_g000050.1:-74..677&addFeatures=%5b%7b%22seq_id%22:%22Rmu_sc0002759.1_g000050.1%22,%22start%22:20,%22end%22:583,%22name%22:%22unnamed%20protein%20product%20Blast%20Hit%22,%22subfeatures%22:%5b%7b%22start%22:20,%22end%22:583,%22strand%22:%221%22,%22type%22:%22match_part%22%7d%5d%7d%5d&addTracks=%5b%7b%22label%22:%22blast%22,%22key%22:%22BLAST%20Result%22,%22type%22:%22JBrowse/View/Track/HTMLFeatures%22,%22store%22:%22url%22%7d%5d) | AT1G64280 | 37.48% | | nonexpresser of PR genes 1 |
| AOS | [Rmu_sc0001670.1](https://www.rosaceae.org/jbrowse/index.html?data=data/rosa/rmultiflora_v1.0&loc=Rmu_sc0001670.1_g000032.1:-86..612&addFeatures=%5b%7b%22seq_id%22:%22Rmu_sc0001670.1_g000032.1%22,%22start%22:1,%22end%22:525,%22name%22:%22unnamed%20protein%20product%20Blast%20Hit%22,%22subfeatures%22:%5b%7b%22start%22:1,%22end%22:525,%22strand%22:%221%22,%22type%22:%22match_part%22%7d%5d%7d%5d&addTracks=%5b%7b%22label%22:%22blast%22,%22key%22:%22BLAST%20Result%22,%22type%22:%22JBrowse/View/Track/HTMLFeatures%22,%22store%22:%22url%22%7d%5d) | AT5G42650 | 65.07% | | allene oxide synthase |
| MYC2 | [Rmu_sc0002616.1](https://www.rosaceae.org/jbrowse/index.html?data=data/rosa/rmultiflora_v1.0&loc=Rmu_sc0002616.1_g000020.1:-146..1027&addFeatures=%5b%7b%22seq_id%22:%22Rmu_sc0002616.1_g000020.1%22,%22start%22:1,%22end%22:880,%22name%22:%22unnamed%20protein%20product%20Blast%20Hit%22,%22subfeatures%22:%5b%7b%22start%22:362,%22end%22:880,%22strand%22:%221%22,%22type%22:%22match_part%22%7d,%7b%22start%22:1,%22end%22:359,%22strand%22:%221%22,%22type%22:%22match_part%22%7d%5d%7d%5d&addTracks=%5b%7b%22label%22:%22blast%22,%22key%22:%22BLAST%20Result%22,%22type%22:%22JBrowse/View/Track/HTMLFeatures%22,%22store%22:%22url%22%7d%5d) | AT1G32640 | 53.03% | | transcription factor MYC2 |
